# Unravelling the mechanism of pressure induced polyamorphic transition in an inorganic molecular glass

**DOI:** 10.1038/s41598-020-61997-x

**Published:** 2020-03-23

**Authors:** Bora Kalkan, Gokce Okay, Bruce G. Aitken, Simon M. Clark, Sabyasachi Sen

**Affiliations:** 1 Earth and Planetary Sciences Department, University of California, Santa Cruz, CA 95064 USA; 2 Advanced Light Source, Lawrence Berkeley National Laboratory, Berkeley, CA 94720 USA; 3 Department of Physics Engineering, Hacettepe University, Ankara, 06800 Beytepe Turkey; 4 Glass Research Division, Corning Inc., Corning, New York, 14831 USA; 5 Department of Earth and Environmental Sciences, Macquarie University, North Ryde, NSW Australia; 6 School of Engineering, Macquarie University, North Ryde, NSW 2109 Australia; 7 Department of Materials Science and Engineering, University of California–Davis, Davis, California 95616 USA

**Keywords:** Glasses, Structure of solids and liquids, Chemical physics

## Abstract

The atomic structure of a germanium doped phosphorous selenide glass of composition Ge_2.8_P_57.7_Se_39.5_ is determined as a function of pressure from ambient to 24 GPa using Monte-Carlo simulations constrained by high energy x-ray scattering data. The ambient pressure structure consists primarily of P_4_Se_3_ molecules and planar edge shared phosphorus rings, reminiscent of those found in red phosphorous as well as a small fraction of locally clustered corner-sharing GeSe_4_ tetrahedra. This low-density amorphous phase transforms into a high-density amorphous phase at ~6.3 GPa. The high-pressure phase is characterized by an extended network structure. The polyamorphic transformation between these two phases involves opening of the P_3_ ring at the base of the P_4_Se_3_ molecules and subsequent reaction with red phosphorus type moieties to produce a cross linked structure. The compression mechanism of the low-density phase involves increased molecular packing, whereas that of the high pressure phase involves an increase in the nearest-neighbor coordination number while the bond angle distributions broaden and shift to smaller angles. The entropy and volume changes associated with this polyamorphic transformation are positive and negative, respectively, and consequently the corresponding Clapeyron slope for this transition would be negative. This result has far reaching implications in our current understanding of the thermodynamics of polyamorphic transitions in glasses and glass-forming liquids.

## Introduction

The existence and the nature of polyamorphic phase transitions in the glassy or liquid state between structurally and thermodynamically distinct phases of the same composition remain controversial issues in the fields of condensed matter physics and chemistry^1^. The most extensively studied case of polyamorphism in the literature pertains to the transitions between the low- and high- density phases of H_2_O in the supercooled liquid state^2–4^. The low-density phase is characterized by an open, hydrogen-bonded tetrahedral “molecular” structure with low entropy, which transforms under pressure into a high-density network structure with high entropy^4^. This inverse relation between volume and entropy implies a negative slope in the P-T space for this polyamorphic transition. However, the structural complexity of water has made the experimental determination of the atomistic mechanism of this structural transformation rather difficult. On the other hand, structurally simple van der Waals -bonded molecular liquids may prove to be more tractable systems for studying the atomistic mechanisms of molecular-to-network polyamorphic transformations.

A handful of inorganic crystals are known to be molecular and consist of nearly spherical cage-like molecules held together by the van der Waals force; examples include As_4_S_3_, P_4_S_3_, P_4_Se_3_ and C_60_. It has been demonstrated in the literature that, amongst these compositions, As_4_S_3_ and P_4_Se_3_ can be glass-forming if the liquid is stabilized against crystallization by the addition of a small concentration of Ge and, in the case of P_4_Se_3,_ by excess phosphorus. These glasses, consisting predominantly of isolated cage molecules, constitute ideal systems for mechanistic studies of molecular-to-network polyamorphic transformation. Recently, we have reported the observation of a pressure-induced polyamorphic transition in a chalcogenide glass of composition Ge_2.5_As_51.25_S_46.25_ at ambient temperature^5–7^. *In situ* high-pressure x-ray scattering, absorption and Raman spectroscopic measurements indicated a hysteretically reversible transformation between a low-density molecular structure and a high-density network structure near ~2 GPa. However, the atomistic mechanism of this structural transformation had remained speculative.

Similar predominantly molecular glasses in the P-Se system near the P_5_Se_3_ composition with and without Ge-doping have been reported in the literature^8–10^. Structural studies of these glasses based on Raman and ^31^P nuclear magnetic resonance (NMR) spectroscopy have indicated the presence of P_4_Se_3_ cage molecules and amorphous red-phosphorus type moieties^8,10^. In this study, we have used a combination of state-of-the art high energy x-ray diffraction and 3D Monte Carlo structural modeling to investigate the structure of such a glass of composition Ge_2.8_P_57.7_Se_39.5_ (denoted henceforth as GPS, for the sake of brevity) and its pressure-dependent evolution *in situ*. The primary goal of this comprehensive study is to obtain a direct understanding of the short and intermediate -range structural aspects and the atomistic mechanism of the pressure-induced molecular-to-network transformation in this glass.

## Results

### Evolution of the structure factor S(Q) at pressures ranging between 0.3 GPa and 24 GPa

**Figure 1:**
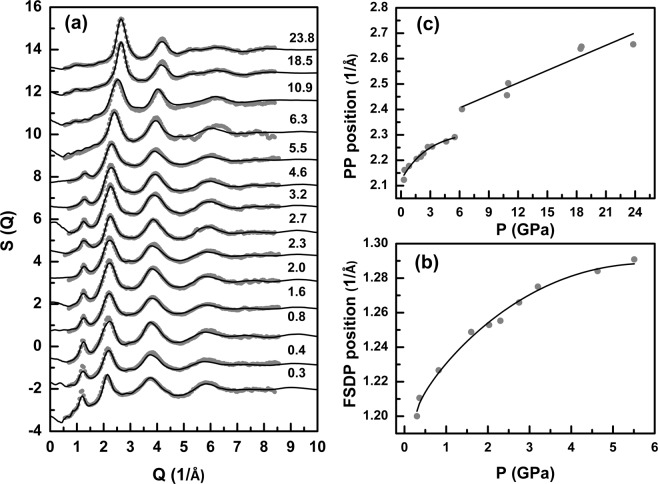
High pressure x-ray scattering data. (**a**) Evolution of S(Q) during compression. Experimental (thick gray dots) and EPSR simulated (black lines) total structure factors at different pressures. Data are vertically offset to enhance clarity and corresponding pressures in GPa are given alongside each pattern. (**b**) Pressure dependence of the position of the FSDP. (**c**) Pressure dependence of the position of the PP in the S(Q). Solid lines through the data points in (**b**) and (**c**) are guides to the eye.

The evolution of the S(Q) of GPS glass with pressure upon compression up to 23.8 GPa is shown in Fig. 1a. The S(Q) is characterized by an intense first sharp diffraction peak (FSDP), located at Q ~ 1.18 Å^−1^ signifying intermediate range order at a length scale of ~5.3 Å, resulting from strong intermolecular correlations in these glasses^11,12^. The chemical and topological ordering in the structure is characterized by the principal peak (PP) and the third peak, respectively^13^. One of the most dramatic changes in the total experimental S(Q) with increasing pressure is a rapid decrease in the intensity of the FSDP. In fact, the FSDP practically disappears completely at pressures above 6.3 GPa (Fig. 1b). During decompression from 23.8 GPa, a hysteretic behavior is observed as the FSDP reappears only when the sample is fully decompressed (Supplementary Fig. S1), although the original intensity of the uncompressed sample is never recovered. On the other hand, the FSDP position rapidly increases to higher Q with pressure in the low-pressure regime below ~1.5 GPa and the slope abruptly changes at higher pressures with a slower rate of increase above ~1.5–2.0 GPa (Fig. 1b). In contrast with the behavior of the FSDP, the intensity of the PP increases with increasing pressure (Fig. 1c) and its position shifts to higher Q values. The PP shifts from ~2.10 Å^−1^ at ambient pressure to ~2.65 Å^−1^ at 23.8 GPa and its intensity increases by a factor of ~2.4. A closer look at the pressure dependence of the PP position shows that the PP position rapidly increases to higher Q with pressure in the low-pressure regime below ~2.5 to 3.0 GPa, followed by a slower rate of increase at higher pressures up to ~6.3 GPa. Further increase in pressure results in an abrupt jump in PP position to 2.4 Å^−1^ at ~6.3 GPa followed by a smooth and monotonic increase in PP position between ~6.3 and 23.8 GPa.

### Structure analysis in real space under pressure

**Figure 2:**
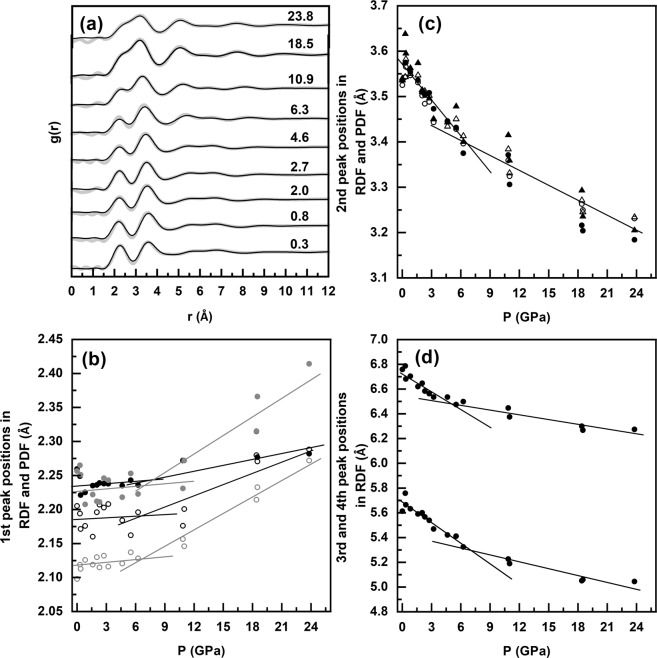
Pressure dependence of RDF. (**a**) Pressure dependence of experimental (gray trace) and simulated (black lines) g(r) during compression of the GPS glass. Data are vertically offset for clarity and corresponding pressures in GPa are given alongside each pattern. (**b**) Pressure dependence of the first peak positions in total g(r) (black dots), and in the P-P (black open circles), P-Se (gray dots), and Ge-Se (gray open circles) PDFs. (**c**) The second peak positions in total g(r) (black dots) and in the P-P (black solid triangles), P-Se (black open triangles), and Se-Se (black open circles) PDFs. (**d**) The third and fourth peak positions in total g(r).

The experimental and simulated radial distribution functions (RDFs) obtained during compression of the GPS glass are compared in Fig. 2a. Constrained EPSR g(r) data enable a rigorous and comprehensive interpretation of short- and intermediate- range structural evolution with pressure. It is clear from Fig. 2a that the qualitative appearance of the RDFs in the high-pressure regime (>6.3 GPa) are distinctly different from those in the low-pressure regime (<6.3 GPa). The pressure dependence of all of the peaks in the RDFs and the corresponding Ge−Se, Se−Se, P-Se and P-P pair distribution functions (PDFs) (Supplementary Fig. S2) determined from the EPSR simulations (Fig. 2b–d) indicates that the intermolecular correlation peaks rapidly shift to shorter distances up to ~6.3 GPa (Fig. 2d). This result suggests that the densification mechanism in this pressure range is dominated by increased molecular packing^14^. The rate of this pressure-induced shift in the peak positions abruptly changes with a slower rate of decrease at pressures above 6.3 GPa. In contrast, the average nearest-neighbor distance in g(r), and the P-Se, P-P and Ge-Se nearest-neighbor peaks in the EPSR PDFs exhibit a slight elongation over the entire pressure range (Fig. 2b). The g(r) curves of GPS glass obtained upon decompression are depicted in Supplementary Fig. S3a. A closer look at the g(r) patterns indicate that the disordered phase obtained after the compression-decompression cycle is somewhat denser than the starting phase (Supplementary Fig. S3b) with slightly shorter intermolecular correlation distances.

**Figure 3:**
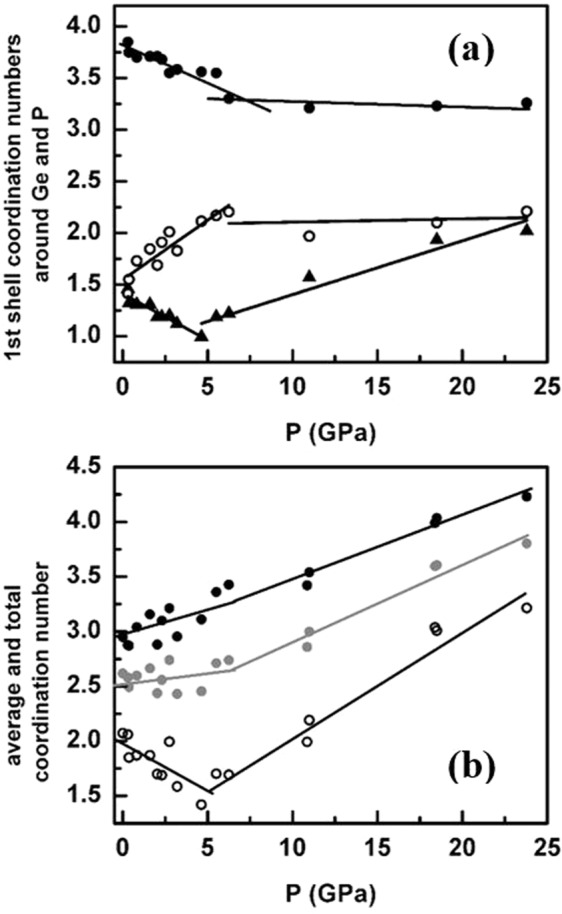
Coordination environments of Ge, P, and Se atoms. (**a**) Pressure dependence of the 1^st^ shell coordination numbers, n_GeSe_ (black dots), n_PP_ (open circles), and n_PSe_ (triangles) obtained from EPSR simulations. (**b**) The average (gray dots) and the 1^st^ shell total coordination numbers around P (black dots) and Se atoms (open circles) as a function of pressure. The lines and curves through the data points in (**a**) and (**b**) are guides to the eye only.

The pressure dependence of the nearest-neighbor coordination numbers n_GeSe_, n_PP_, and n_PSe_, the total coordination numbers around P and Se, and the average first shell coordination numbers calculated from the EPSR simulation are shown in Fig. 3a,b. According to the 8-N rule, the coordination numbers of Ge, Se and P atoms are expected to be 4, 2 and 3, respectively, which would lead to an average first shell coordination number, n_average_ of 2.63 for the GPS composition. The results of the EPSR-simulated structure indicate that n_average_ for the GPS glass at ambient pressure is ~2.64. The average value of n_GeSe_ is ~3.7, which however, is somewhat lower than 4.0 for tetrahedral coordination. The total coordination number around the P atoms is calculated using first shell P-P and P-Se correlations and found to be ~3.10, while that for the Se atoms, calculated using first shell Se-Ge and Se-P correlations yields a value of ~2.10. The results indicate that n_average_ does not change significantly up to ~5.5 GPa. Further increase in pressure results in a rapid increase in n_average_ to 3.8 at 23.8 GPa (Fig. 3b). This corresponds to a ~46% increase of n_average_ in the pressure range of 5.5 to 23.8 GPa. The average value of n_GeSe_ shows an unusual behavior where it decreases from 3.7 to 3.3 between ambient and 6.3 GPa (Fig. 3a) and then remains practically constant above 6.3 GPa. A similar behavior is observed for n_PSe_, which decreases from ~1.5 typical of P_4_Se_3_ molecules at ambient pressure to ~1.0 at 6.3 GPa and then the trend abruptly reverses with progressive increase above 6.3 GPa and n_PSe_ reaches ~2.0 at 23.8 GPa (Fig. 3a). On the other hand, n_PP_ increases from ~1.4 at ambient pressure, as expected for P_4_Se_3_ molecules, to ~2.2 at 6.3 GPa such that the total P coordination number at remains ~3.0 at pressures below 6.3 GPa. Further increase in pressure results in almost a nearly invariant n_PP_ up to 23.8 GPa such that the total P coordination number becomes ~4.0 at the highest pressures (Fig. 3b). The lowering of n_PSe_ and n_GeSe_ with pressure between ambient and 6.3 GPa is reflected in a drop in the total coordination number for Se from ~2.1 at ambient to ~1.5 near 6.3 GPa. At higher pressures the densification ultimately results in the formation of ~3- and ~4- fold coordinated Se and P atoms, respectively, at 23.8 GPa.

**Figure 4:**
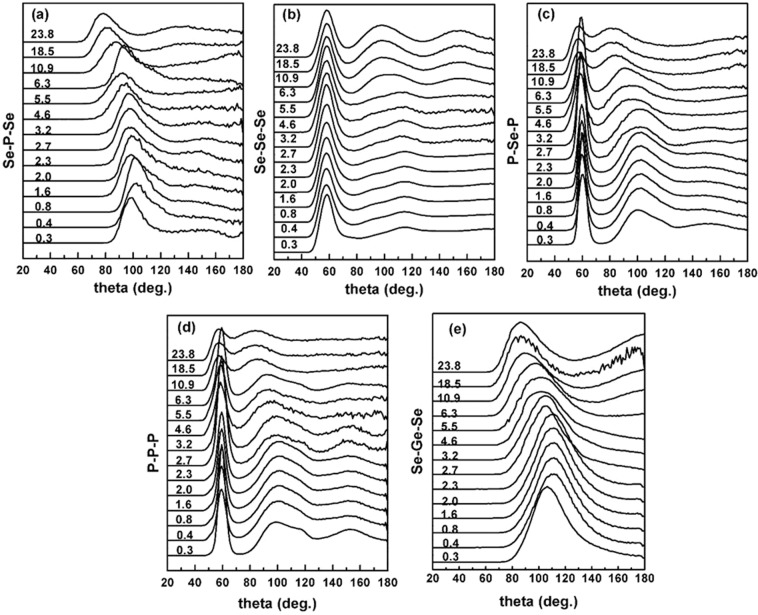
Pressure dependence of bond angle distributions. Pressure dependence of P−P−P (1^st^ shell), P−Se−P (1^st^ shell), Se-P-Se (1^st^ shell), Se-Ge-Se (1^st^ shell) and Se-Se-Se (2^nd^ shell) bond angle distributions obtained from the EPSR simulations. Data are vertically offset for clarity and corresponding pressures in GPa are given alongside.

The pressure dependence of these various intra- and inter- molecular bond angles is shown in Fig. 4. Again, it is clear from Fig. 4 that the evolution of these bond angles is distinctly different in the low-pressure regime (<6.3 GPa) compared to that in the high-pressure regime (>6.3 GPa). As the pressure increases the Se-P-Se BAD (arises from P_4_Se_3_ molecules where the apical P atom is linked to three Se atoms and formed PSe_3_ pyramids) and Se-Ge-Se BAD (GeSe_4_ tetrahedral angle) broaden and shift to smaller angles. The rate of this shift increases abruptly at pressures >6.3 GPa. The intra-molecular P-Se-P BAD peak centered at ~100° (arises from the linkage of apical P atom to basal P_3_ plane via P-Se bonds) displays a similar behavior. The broad peak in the P-P-P distribution (centered in the range of ~80–130°) corresponding to the amorphous red-P type structural moieties also displays a rapid shift to lower angles with pressure above 6.3 GPa.

**Figure 5:**
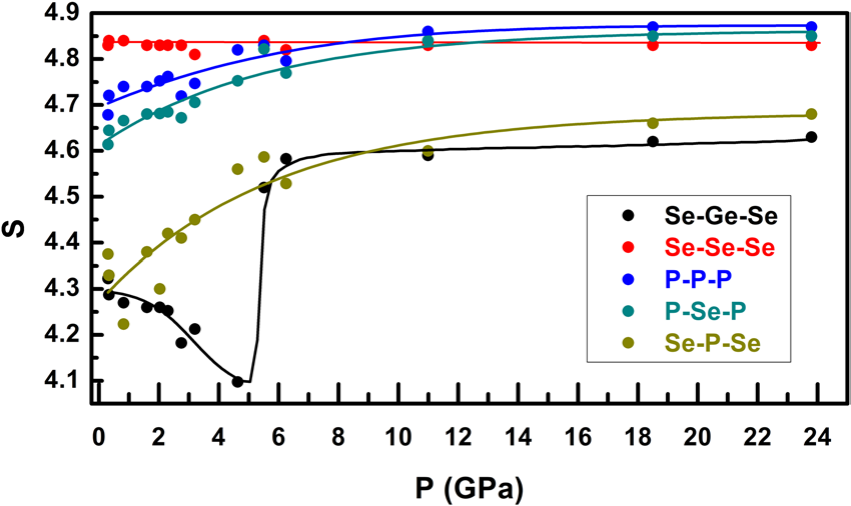
Entropy Information entropy of the bond angle distributions.

The information entropy of the bond angle distribution functions is calculated using ${\boldsymbol{S}}=-\,{\boldsymbol{\sum }}_{{\boldsymbol{i}}}{{\boldsymbol{P}}}_{{\boldsymbol{i}}}\,{\boldsymbol{ln}}\,{{\boldsymbol{P}}}_{{\boldsymbol{i}}}$, where *P*_*i*_ is the distribution of the bond angles. The entropy of the bond angle distribution function is a measure of how well defined the bond angles are at each pressure (large entropy, broad distribution). The pressure dependence of the entropy is depicted in Fig. 5. A very dramatic change in the entropy up to 6.3 GPa is observed for Se−Ge−Se and Se-P-Se bond angle distributions, rather than the P−P−P, P−Se−P distributions, implying a fast structural change in the atomic configuration. For each distribution, above 6.3 GPa, the curve shows a change in slope, and the growth in S is slower. Se-Se-Se distribution calculated for second shell neighborhood distances does not alter the information entropy, which implies the stability of Se distribution in the second shell neighborhood. However, it must be noted that only a part of the configurational entropy is associated with that of the bond angle distributions. Therefore, a direct one-to-one correlation between the pressure dependence of the bond angle entropy and that of the degree of molecular-to-network transformation may not be obvious. Nevertheless, the abruptness in the change in the Se-Ge-Se bond angle distribution entropy is the most distinct among the order parameters and indicates a fundamental shift in the densification mechanism. This result is indeed interesting since the presence of GeSe_4_ clusters is crucial to the glass-forming ability of this molecular liquid.

### Distortion of GeSe_4_ tetrahedral units and PSe_3_ pyramids in P_4_Se_3_ molecules

**Figure 6:**
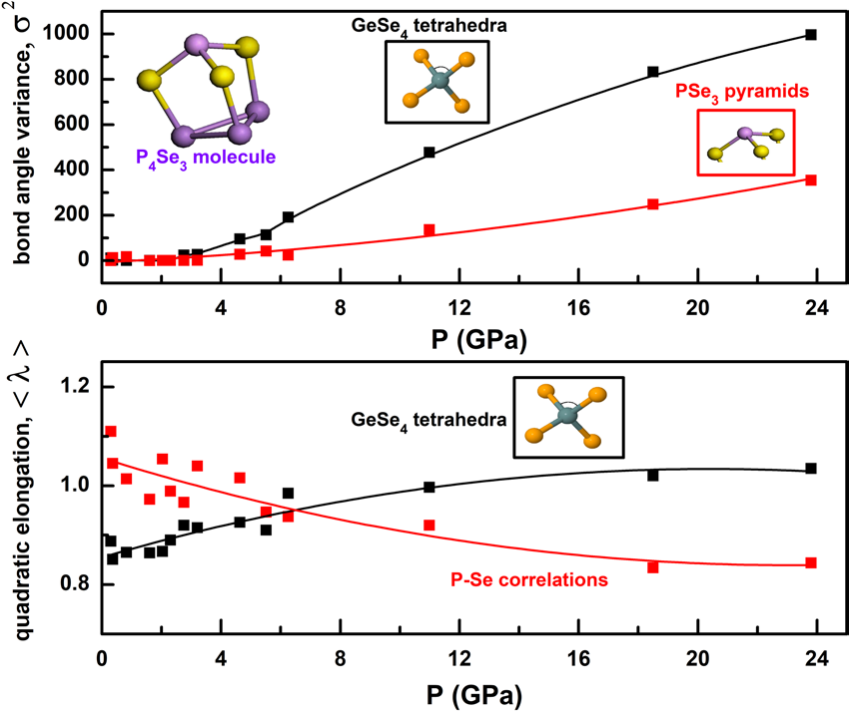
Pressure variation of distortion parameters. Top: Variation of bond-angle variance with pressure for GeSe_4_ tetrahedral units and PSe_3_ pyramids in P_4_Se_3_ molecules. Bottom: Pressure dependence of the quadratic elongation of GeSe_4_ and intra- and inter- molecular P-Se correlations.

The degree of distortion for all molecules is quantified in terms of quadratic elongation (QE) and angle variance (AV). The former gives a measure of the distortion in bond lengths which would all be equal in an ideal polyhedron whereas the latter gives a measure of the distortion of bond angles from their ideal values. The ideal values of QE and AV are unity and zero, respectively. The results are depicted in Fig. 6. QE values have been calculated using the average Ge-Se and P-Se bond-lengths where the former has contribution from GeSe_4_ tetrahedral units only but the latter gives the distortion in both P_4_Se_3_ intra- and inter- molecular bond lengths. QE results (Fig. 6) suggest the instability of the GeSe_4_ tetrahedral units. As pressure rises, the degree of distortion slightly increases for GeSe_4_ tetrahedral units but QE values for P_4_Se_3_ intra- and inter- molecular bonding structure decreases. This can be related to the fact that the P-Se correlations include more complex structure, covering both inter-molecular and intra-molecular P-Se bonds. AV results (Fig. 6), which have been calculated using the Se-Ge-Se (inter-GeSe_4_ tetrahedral) and Se-P-Se (PSe_3_ pyramids in P_4_Se_3_ molecules) bond angle distributions, give us a better understanding about the degree of distortion. The GeSe_4_ tetrahedral shows a much larger distortion level (above 6 GPa) than PSe_3_ pyramids do. GeSe_4_ units remain the most distorted over the pressure range studied.

## Discussion

The S(Q) and g(r) at ambient pressure in combination with the EPSR simulation show interatomic distances, coordination numbers, various bond angles and PDFs that are all consistent with a structural scenario that is in excellent agreement with previous studies on this and similar glasses based on ^31^P NMR and Raman spectroscopy. In this scenario, the GPS glass structure at ambient pressure consists predominantly of P_4_Se_3_ molecules and amorphous red-phosphorus type moieties along with a small concentration of GeSe_4_ tetrahedra^15^. The bonding of the constituent elements appears to largely obey the 8−N rule. The relative concentrations of these structural units can be estimated by considering that the GPS glass composition can be written as: (GeSe_2_)_2.8_ + (P_4_Se_3_)_11.3_ + (P)_12.5_. The lack of any Ge-P nearest-neighbor and Ge-P-Se angular correlations (Supplementary Fig. S2) is indicative of the absence of any significant cross-linking between molecules via Ge atoms. Rather, the presence of a broad but distinct Ge-Se-Ge correlation suggests local clustering of corner-shared GeSe_4_ tetrahedra in the glass structure. This clustering of Ge atoms is also evident in Supplementary Fig. S4a, showing the spatial distribution of Ge atoms in a part of the simulation cell. On the other hand, local structural motifs taken from the simulation cell show evidence for amorphous red-phosphorus type moieties enabling cross-linking between a small fraction of P_4_Se_3_ molecules via an opening of their basal P_3_ ring (Supplementary Fig. S4b).

When taken together, the pressure dependent structural evolution of the S(Q), G(r) and all structural parameters obtained from EPSR simulation yield a consistent scenario of a (predominantly) molecular-to-network polyamorphic structural transformation of the GPS glass across ~6.3 GPa. In the low-pressure regime between ambient and 6.3 GPa, the structure remains predominantly molecular and densifies via increased molecular packing which is initially relatively rapid up to ~2–3 GPa and subsequently slows down at higher pressures up to ~ 6.3 GPa, presumably due to increased intermolecular repulsion. This change in molecular packing rate is evident in the pressure dependence of the positions of the FSDP and the PP (Fig. 1b,c). Increased molecular packing also results in a loss of the intermediate-range ordering associated with inter-molecular correlations which is manifested in the rapid lowering of the intensity of FSDP in this pressure regime. It is rather intriguing to note that, besides increased molecular packing, the P-P coordination number increases from ~1.5 to ~2.0 in this pressure regime (Fig. 3a). This result indicates the onset of cage opening for a significant fraction of the P_4_Se_3_ molecules via breaking of the P-Se-P intramolecular linkages between the apical PSe_3_ pyramid and the basal P_3_ triangle. This observation is consistent with the results reported in previous *in situ* high-pressure Raman spectroscopic studies of the Ge_2.5_As_51.25_S_46.25_ glass with As_4_S_3_ cage molecules that demonstrated complete disappearance of the breathing mode of the basal As_3_ triangle near ~3 GPa^7^. Once a critical fraction of the molecules undergoes cage opening, the structure collapses into a network near ~6.3 GPa with the simultaneous loss of the FSDP and an abrupt shift in the PP position (Fig. 1c). The densification of this network in the high-pressure regime is marked by rather abrupt changes in the g(r) and the PDFs as well as in the rate of change of various bond lengths and angles (Figs. 2, 4). Network structures typically densify at high pressures via increase in the coordination numbers of the constituent atoms. This phenomenon is evident in the behavior of the coordination numbers of P and Se atoms in the GPS glass, which increase in the high-pressure regime to 4 and 3 (from 3 and 2), respectively, at ~23.8 GPa (Fig. 3b).

**Figure 7:**
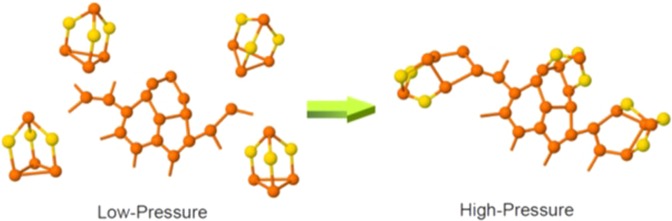
Cage-opening mechanism in GPS glass. The cage opening of P_4_Se_3_ molecules as a precursor for the network formation. The polyamorphic transformation between low pressure and high pressure structures involves opening of the P_3_ ring at the base of the P_4_Se_3_ molecules and subsequent reaction with planar edge-shared phosphorus rings.

As noted earlier, a pressure-induced molecule-to-network polyamorphic transformation was also reported in the literature for the Ge_2.5_As_51.25_S_46.25_ glass with As_4_S_3_ cage molecules^5,7^. However, these studies did not directly address the structural mechanism for this transformation. The results obtained in the present study clearly indicate the cage opening of P_4_Se_3_ molecules as a precursor for the network formation. This cage-opening mechanism is schematically shown in Fig. 7. Furthermore, to the best of our knowledge, this is the first report of clear observation of two distinct pressure regimes for the densification of the molecular and network structures which puts polyamorphism in these molecular systems on a firm footing. It is clear that the low pressure molecular structure is more ordered compared to high pressure network structure counterpart, which indicates positive entropy and negative volume changes associated with the transformation. Consequently, the slope of the P-T phase boundary should be negative. This hypothesis would imply that the transition could be observed at ambient pressure upon increasing temperature. Future measurements of the viscosity of the Ge_2.5_As_51.25_S_46.25_ liquid over a wide temperature range may reveal the presence of such transformations in the equilibrium liquid state. It is to be noted that recent studies indeed suggest a liquid-liquid phase transition at ambient pressure with increasing temperature can be observed in the form of a sudden change in the temperature dependence of viscosity^16–18^. In fact, the recent proposal of possible strong-to-fragile transitions in glass-forming liquids with increasing temperature^18^ may be connected to their pressure-dependent counterparts in polyamorphic transitions with negative P-T slopes.

## Methods

### Sample preparation

The GPS glass was synthesized by melting a mixture of the starting elements (≥99.9995% purity in metals basis) in an evacuated (10^−6^ Torr) fused silica ampoule at 873 K for at least 13 h in a rocking furnace. The ampoule was rapidly quenched in water to make transparent orange-red glass. The glass sample was confirmed to be amorphous using powder x-ray diffraction. The composition reported above was obtained from electron probe micro-analysis. The small amount of Ge doping and P-excess (over the P_4_Se_3_ stoichiometry) are necessary in stabilizing this glass against devitrification. The density (3.09 gcm^−3^), glass transition temperature T_g_ (468 K), Raman and ^31^P NMR spectra of this glass were reported in a previous publication^8^.

### X-ray diffraction and absorption

High pressure/room temperature (hprt-XRD) x-ray diffraction data were collected on beamline 12.2.2 at the Advanced Light Source (ALS)^19^. An x-ray energy of 30 keV (λ = 0.4133 Å) was selected to collect the hprt-XRD data. A single x-ray wavelength was selected using a Si(111) double crystal monochromator (12.2.2, ALS). The x-ray beam was focused to a 10 × 10 µm spot size at the sample position. X-ray diffraction images were collected using a MAR345 image plate detector. The sample detector distance and the detector tilt angles were measured using powder diffraction from a LaB_6_ standard. The x-ray beam was 99% horizontally polarized and all geometric and polarization corrections were made during the angular integration using the FIT2D^20^ software package. Details of the x-ray diffraction data reduction process to obtain the structure factors, S(Q) were described in our previous study^21^. A typical process was exemplified in Supplementary Fig. S5 for the x-ray diffraction data collected at 0.4 GPa. For absorption measurements and determination of densities we followed the method described by Shen *et al*.^22^. A rhenium gasket pre-indented to thickness of ~22 µm was used between diamond anvils. Two chambers with ~90 μm diameter were formed by laser-drilling, and serve as cavities for sample material (loaded into one of the holes) and NaCl (loaded into the other hole). Absorption measurements were performed in the pressure range of 0–25 GPa using a 10 µm x-ray beam with the energy of 20 keV (λ = 0.6199 Å). The x-ray intensity transmitted through each hole was measured using a pin-diode with the uncertainty of ±0.05 (see Supplementary Fig. S6). By measuring the transmitted intensities, gasket thickness and sample density have been obtained as 22.1 ± 1.7 μm and 3.09 ± 0.18 g/cm^3^, and 10.2 ± 0.2 μm and 4.66 ± 0.60 g/cm^3^ (see Supplementary Fig. S7 for the full list in 0–25 GPa pressure range) at 0.3 GPa and 25 GPa, respectively. The uncertainties were determined by the errors in the x-ray transmission intensities and in the unit cell volumes obtained by diffraction. The standard errors reported here are (±0.60 g/cm^3^ maximum) relatively bigger than the ones obtained in ref. ^22^. (±0.11 g/cm^3^), which indicates considerable irregularities in transmission profile (Supplementary Fig. S6).

### High pressure generation

High pressures were generated using a symmetric diamond anvil cell (DAC) equipped with 400 µm culet diamonds, c-BN seats and a rhenium gasket pre-indented to a thickness of 27 µm. A 100 µm hole was drilled in the center of the indentation, and then loaded with sample, two spheres of ruby as a pressure marker, and a 4:1 methanol: ethanol mixture as the pressure-transmitting fluid. The ruby fluorescence spectra showed a well resolved doublet for all measurements we performed, which leads us to conclude that the sample is compressed in fairly hydrostatic pressure environment.

### 3D structural modeling

Empirical Potential Structure Refinement (EPSR) method^23^ was used to determine the atomic structure of GPS glass at ambient and high pressure. For each simulation, 56 Ge, 1154 P and 790 Se atoms were mixed in a cubic simulation box with a volume constrained by the experimentally determined glass density (Supplementary Tables S1, S2). The Lennard-Jones potential well depth parameters and range parameters were set to 0.1 kJ/mol and 2.0 Å, 0.5 kJ/mol and 2.2 Å, 0.1 kJ/mol and 2.0 Å for Ge, P and Se, respectively^21,24^. Minimum approach distances listed in (Supplementary Table S1) were used to constrain the modelling of experimental S(Q).

## Supplementary information

Supplementary information.
