# Supplementary material for: Unravelling the mechanism of pressure induced polyamorphic transition in an inorganic molecular glass
Source: Sci Rep. 2020 Mar 23;10:5208. doi: 10.1038/s41598-020-61997-x (PMC7089991; doi:10.1038/s41598-020-61997-x)
Supplement: Supplementary file 1 — Supplementary information. [file 41598_2020_61997_MOESM1_ESM.doc]

***Supplementary Information***

**Unravelling the mechanism of pressure induced polyamorphic transition in an inorganic molecular glass**

Bora Kalkan*^1,2^*,* Gokce Okay^3^, Bruce G. Aitken^4^, Simon M. Clark^5,6^*,* and Sabyasachi Sen^7^

*^1^Earth and Planetary Sciences Department, University of California, Santa Cruz, CA 95064, USA.*

*^2^Advanced Light Source, Lawrence Berkeley National Laboratory, Berkeley, CA 94720, USA.*

*^3^Department of Physics Engineering, Hacettepe University, Ankara, 06800 Beytepe, Turkey. .*

*^4^Glass Research Division, Corning Inc., Corning, New York 14831, USA.*

*^5^Department of Earth and Environmental Sciences, Macquarie University, NSW, 2109 Australia.*

*^6^School of Engineering, Macquarie University, North Ryde, NSW, 2109 Australia.*

*^7^****Department of Materials Science and Engineering, University of California–Davis, Davis, California 95616, USA.***

| Table S1 | Parameters used to build the simulation box.* | | | |
| --- | --- | --- | --- |
| 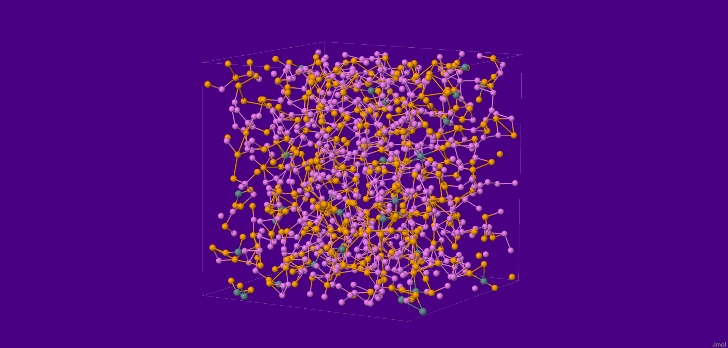  Grey atoms: Ge, Orange atoms: Se, Purple atoms: P | | | |
| Pairs | Minimum approach distances (Å) | | |
| Ge-Ge | 2.80 | | |
| Ge-Se | 1.60 | | |
| P-P | 2.16 | | |
| P-Se | 2.17 | | |
| Ge-P | 2.80 | | |
| Se-Se | 3.00 | | |
| Glass | Mass density (g/cm^3^) | Atomic number density (atoms/ Å^3^) | |
| GPS | 3.09 | 0.0350 | |
| Element | Number of atoms(GPS) | Ɛ (kJ/mol) | σ (Å) |
| Ge | 56 | 0.10 | 2.00 |
| P | 1154 | 0.50 | 2.20 |
| Se | 790 | 0.10 | 2.00 |
| *Mass and atomic number densities, minimum approach distances, number of atoms, and Lennard-Jones parameters used in EPSR simulations. | | | |

| Table S2| Pressure dependence of mass densities obtained from absorption measurements. | | |
| --- | --- | --- |
| Ge_2.8_P_57.7_Se_39.5_ |  |  |
| P (GPa) | Mass density (gcm^-3^) | |
| 0.3 | 3.09(18) | |
| 0.5 | 3.44(19) | |
| 1.1 | 3.29(23) | |
| 2.0 | 3.46(25) | |
| 2.7 | 3.44(28) | |
| 3.5 | 3.53(30) | |
| 4.1 | 3.51(35) | |
| 6.8 | 3.70(39) | |
| 9.6 | 3.83(41) | |
| 14.0 | 4.04(45) | |
| 19.2 | 4.36(46) | |
| 21.0 | 4.50(49) | |
| 22.6 | 4.58(52) | |
| 24.0  25.0 | 4.61(56)  4.66(60) | |

| Table S3| The atomic number and mass densities used in EPSR simulations. | | |  |
| --- | --- | --- | --- |
| Ge_2.8_P_57.7_Se_39.5_ |  |  |  |
| P (GPa) | Atomic number density (atoms/ Å^3^) | | Mass density (gcm^-3^) |
| 0.3 | 0.0350 | | 3.090 |
| 0.4 | 0.0390 | | 3.443 |
| 0.8 | 0.0370 | | 3.266 |
| 1.6 | 0.0390 | | 3.441 |
| 2.0 | 0.0390 | | 3.444 |
| 2.3 | 0.0393 | | 3.469 |
| 2.7 | 0.0394 | | 3.478 |
| 3.2 | 0.0399 | | 3.523 |
| 4.6 | 0.0403 | | 3.558 |
| 5.5 | 0.0410 | | 3.619 |
| 6.3 | 0.0415 | | 3.664 |
| 10.9 | 0.0440 | | 3.885 |
| 18.5 | 0.0490 | | 4.326 |
| 23.8 | 0.0520 | | 4.591 |

**Figure S1.** Experimental (thick gray bars) and EPSR simulated (black thin lines) total structure factors of GPS glass obtained upon decompression. Data are vertically offset to enhance clarity and corresponding pressures in GPa are given alongside each pattern.

**
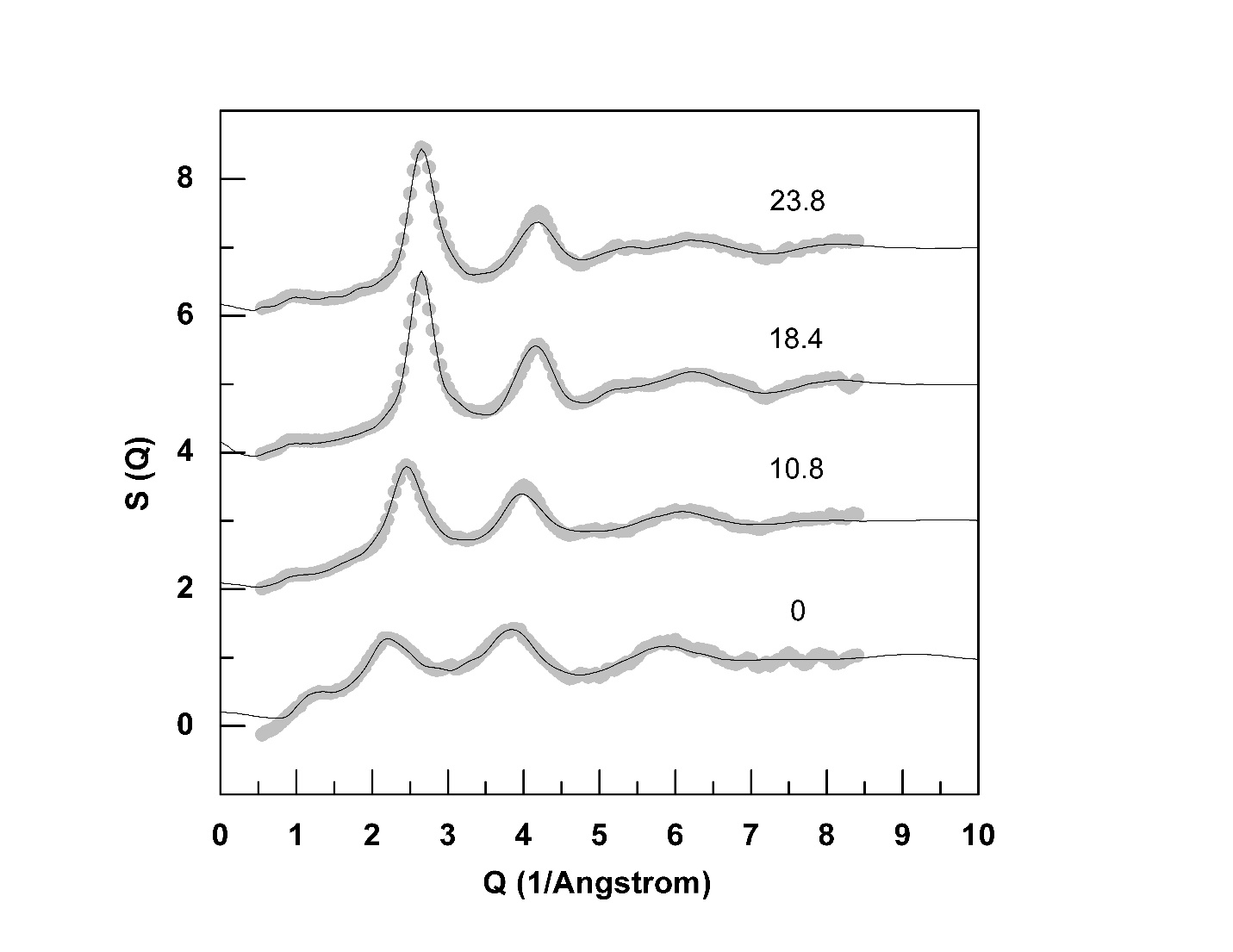
**

**Figure S2. X-ray scattering data and structure of GPS glass in real space at ambient condition.** (**a**) Experimental (gray dots) and EPSR simulated (solid black line) total structure factors S(Q) at ambient pressure and temperature. (**b**) The radial distribution functions g(r) of GPS glass calculated from the EPSR simulations (solid black line) compared with g(r) obtained by Fourier transforming the experimental S(Q) data (gray dots). (**c**) Ge-Se (bottom), P-P (above Ge-Se), P-Se (below Se-Se), and Se-Se (top) PDFs, as obtained from Fourier transformation of the corresponding partial structure factors. (**d**) The ambient BAD functions obtained from the EPSR simulations. The ambient data for S(Q), g(r), PDFs and BAD functions were adapted from our recent study [1].

**
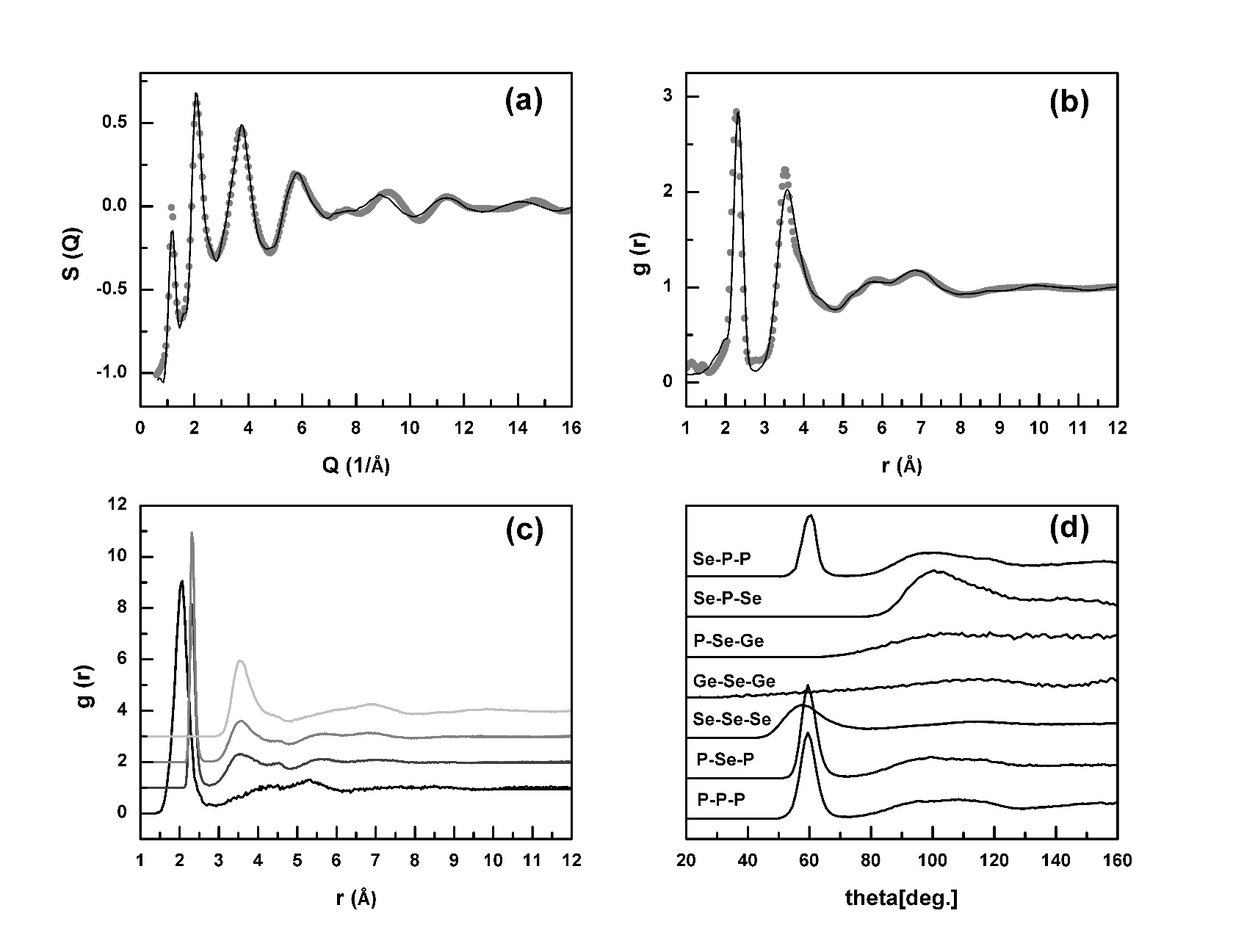
**

**Figure S3.** (**a**) EPSR-simulated g(r) curves of GPS glass upon decompression. Data are vertically offset to enhance clarity and corresponding pressures in GPa are given alongside each pattern. **(b)** RDFs of GPS glass at ambient temperature and before compression at 0.3 GPa (gray) and after complete decompression (black)


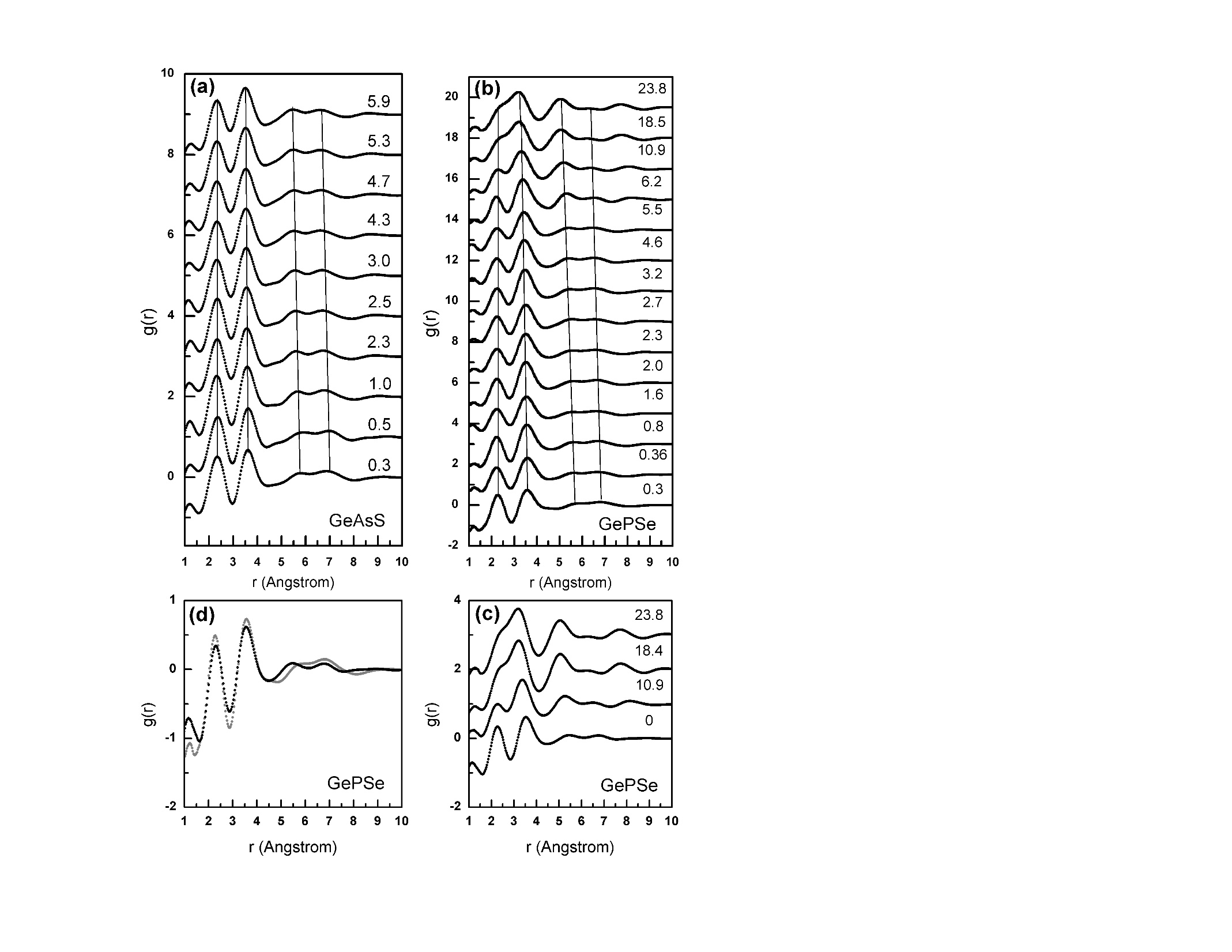

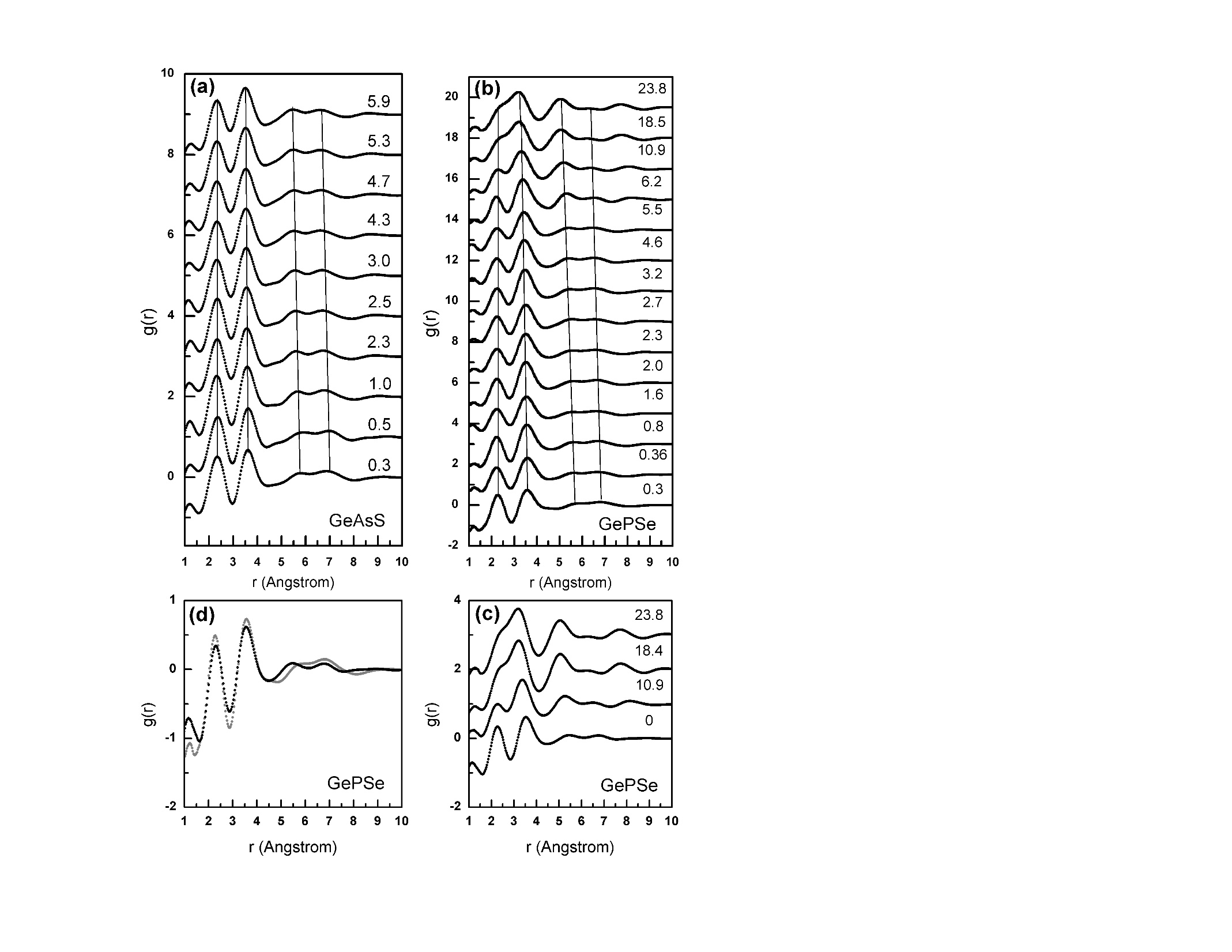


**a**

**b**

**Figure S4 | Structural motifs at ambient pressure captured from EPSR simulation box.** (**a**) Corner-shared GeSe(S)_4_ tetrahedral units in GPS glass. Only Ge and Se atoms are shown. (**b**) Schematic representation of P_4_Se_3_ molecules and amorphous red-phosphorus type moieties in GPS glass.


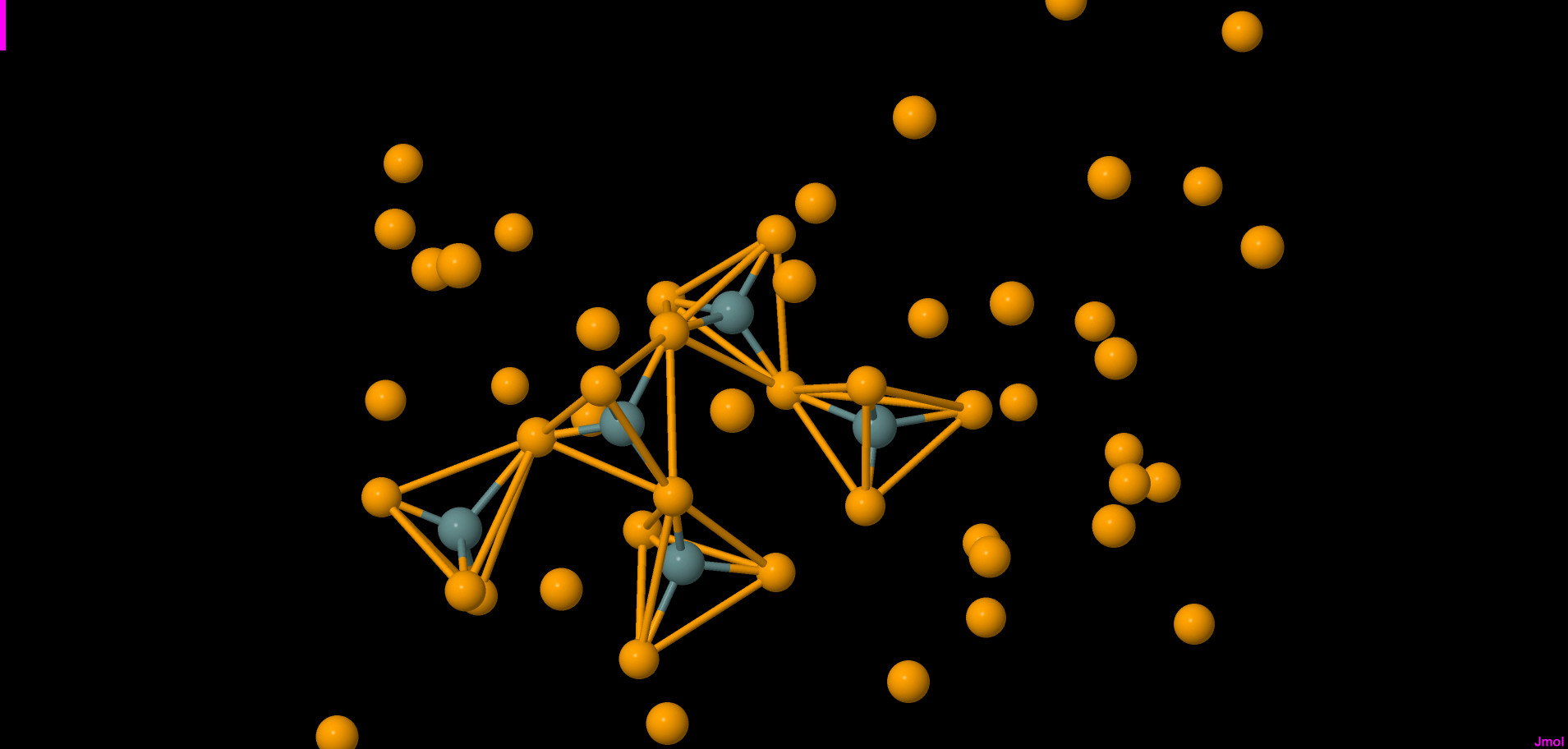

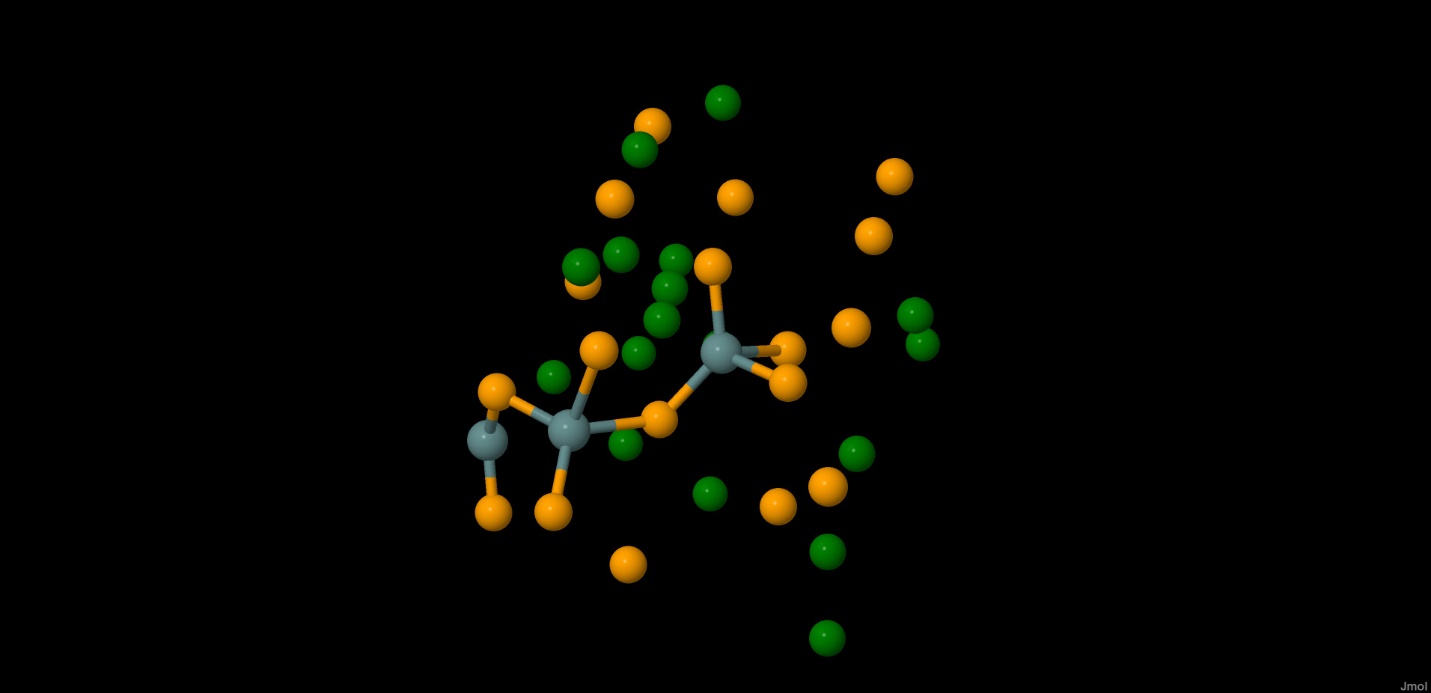

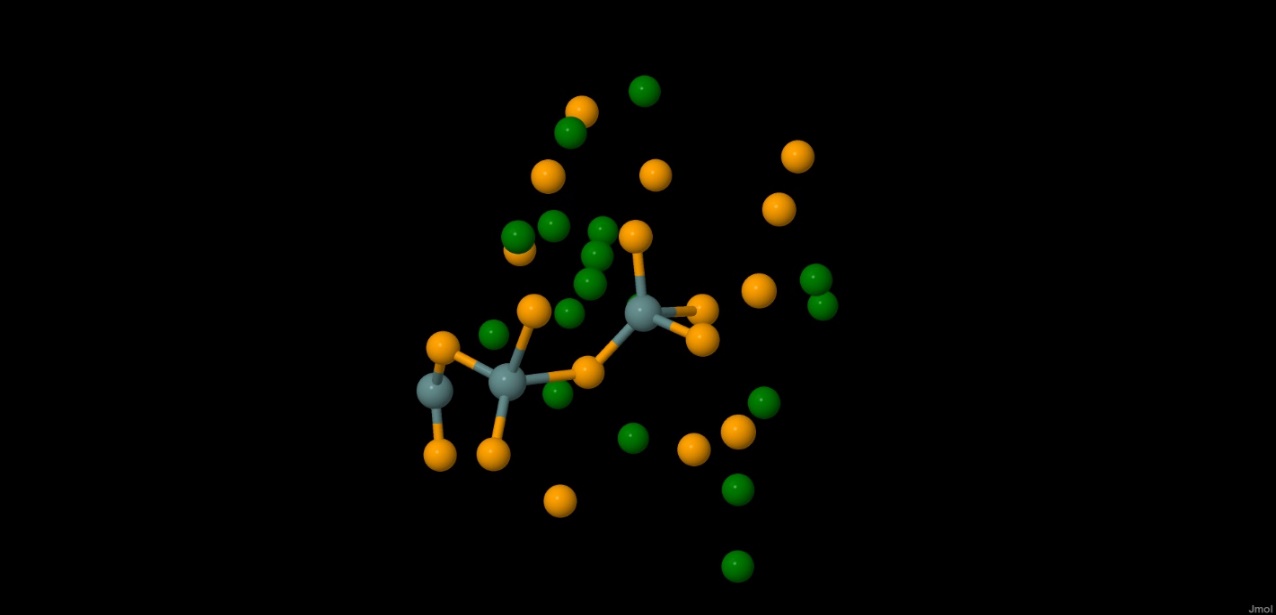

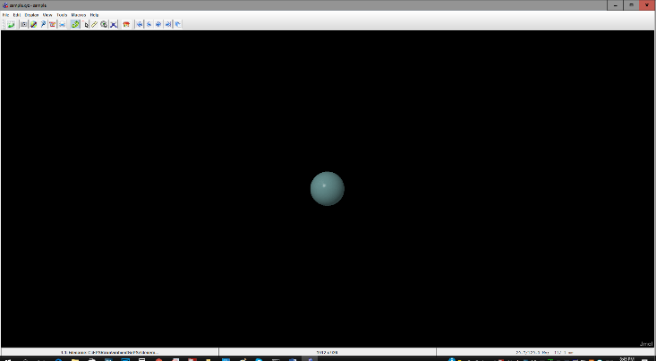


**Se**

**Ge**

**(a)**

**(b)**


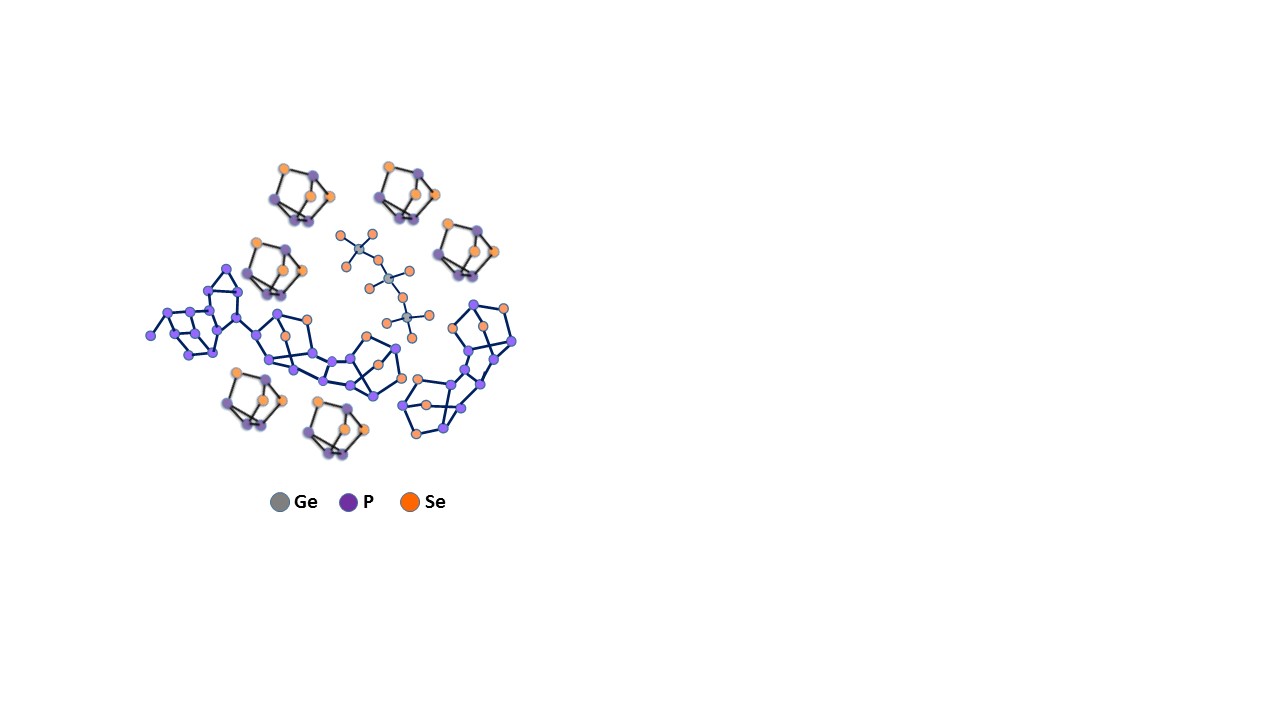


**Figure S5 | Details of the data reduction process to obtain the structure factor from raw diffraction pattern collected at 0.4 GPa.** The raw intensity measured for the sample (black), *I^obs^(Q)* and a diffraction pattern of the background, *I^back^(Q)* recorded through the pressure transmitting medium in sample cavity (orange). The intensity from sample (background corrected), *I^samp^(Q)* (blue) is calculated using the equation of *I^samp^(Q)= I^obs^(Q)-b I^back^(Q),* where *b* is the background correction factor, and equals unity for 0.4 GPa data. The measured intensities were converted into electron units per atom by the generalized Krogh-Moe-Norman method [2] (green), and the coherent x-ray scattering intensity, *I^coh^(Q)* for studied system is expressed by the equation of *I^coh^(Q) = (< f ^2^> - < f > ^2^) + < f > ^2^ S(Q)* in terms of the Faber-Ziman (FZ) notation, where *S(Q)* is the structure factor (inset), and the *< f ^2^ >-< f > ^2^* term is attributed to the intensity arising only from the difference in the atomic scattering factors of the constituent atoms. The scattering factors *< f >^2^* (green) and *<f ^2^>* are determined using the tabulated values, and also note that at high Q region the coherent scattering oscillates around *< f >^2^* (pink).

**
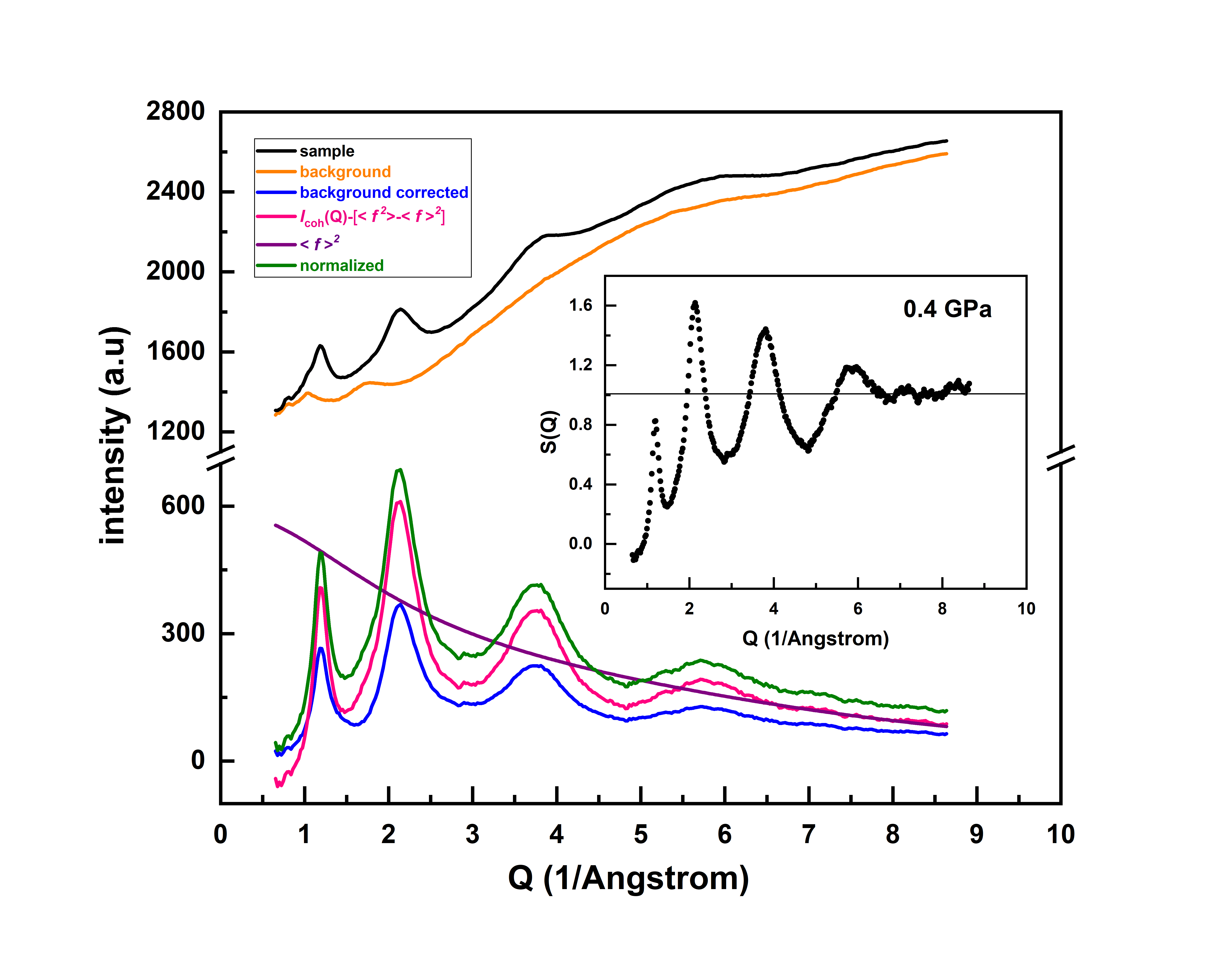
**

**Figure S6 | Transmitted intensity profiles.** The average transmitted intensities for sample (red) and NaCl (black). The contour plot obtained at 0.4 GPa is shown as inset. The step size for the horizontal and vertical scans (Sample X and Y motors) was 5 µm, and 10 µm beam size has been chosen. Transmitted intensity is measured for 1s. The color code indicates that red means high, blue corresponds to lowest intensities. Better transmitted intensity profile and less irregularities can be achieved by using smaller x-ray beam (less than 5 µm in size), longer counting periods, and choosing proper x-ray energy to get better absorption contrast between sample and NaCl.


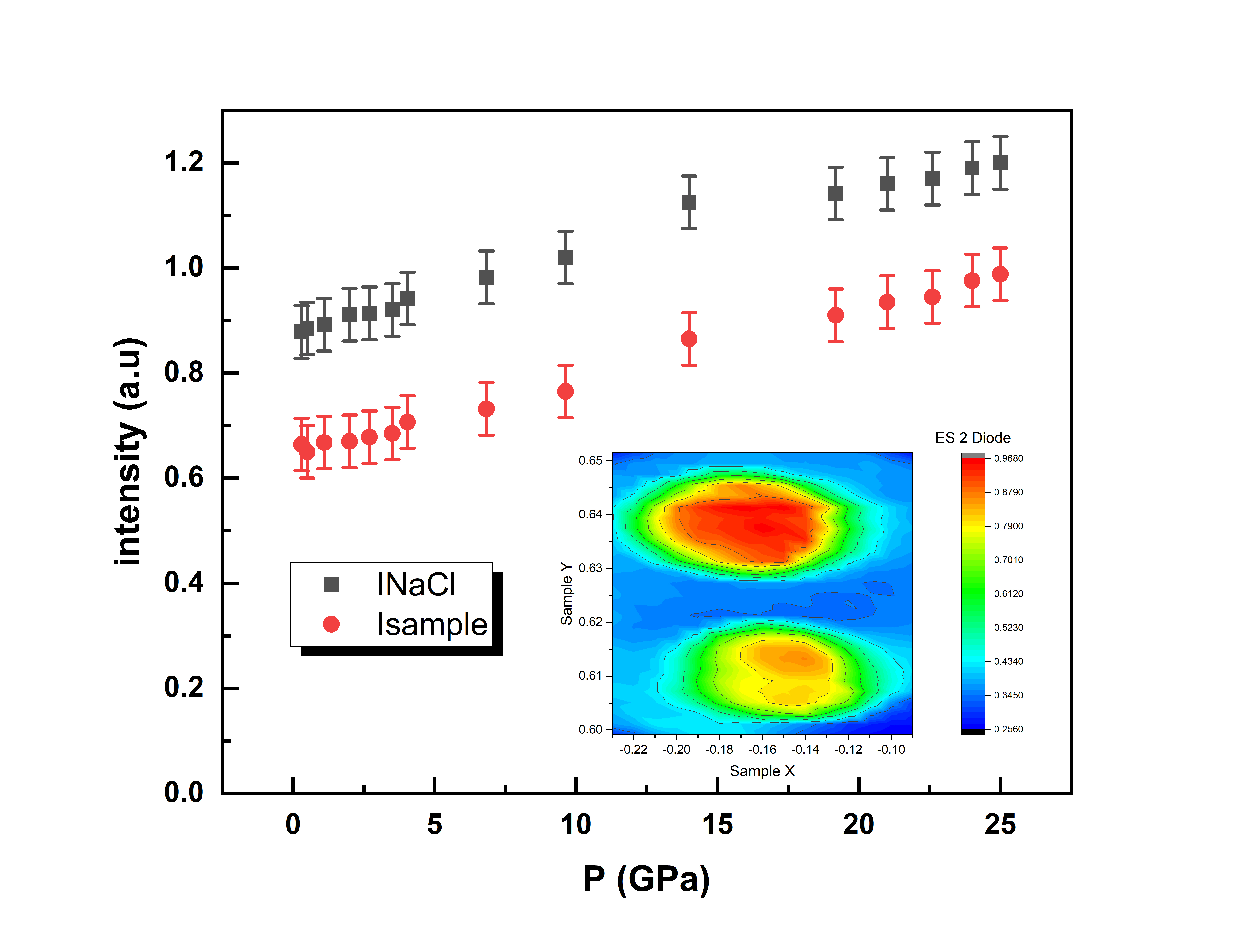


**Figure S7 | Results of the density measurements.** (**a**) Thickness variation of the gasket as a function of pressure. The pressure was determined from the lattice parameters of NaCl. (**b**) Densities of sample at room temperature in 0-25 GPa pressure range (gray dots). Plotted data are also listed in Table S2. The densities used in EPSR simulations (listed in Table S3) are chosen according to the curve tracing experimental values, and are shown with black dots.

**
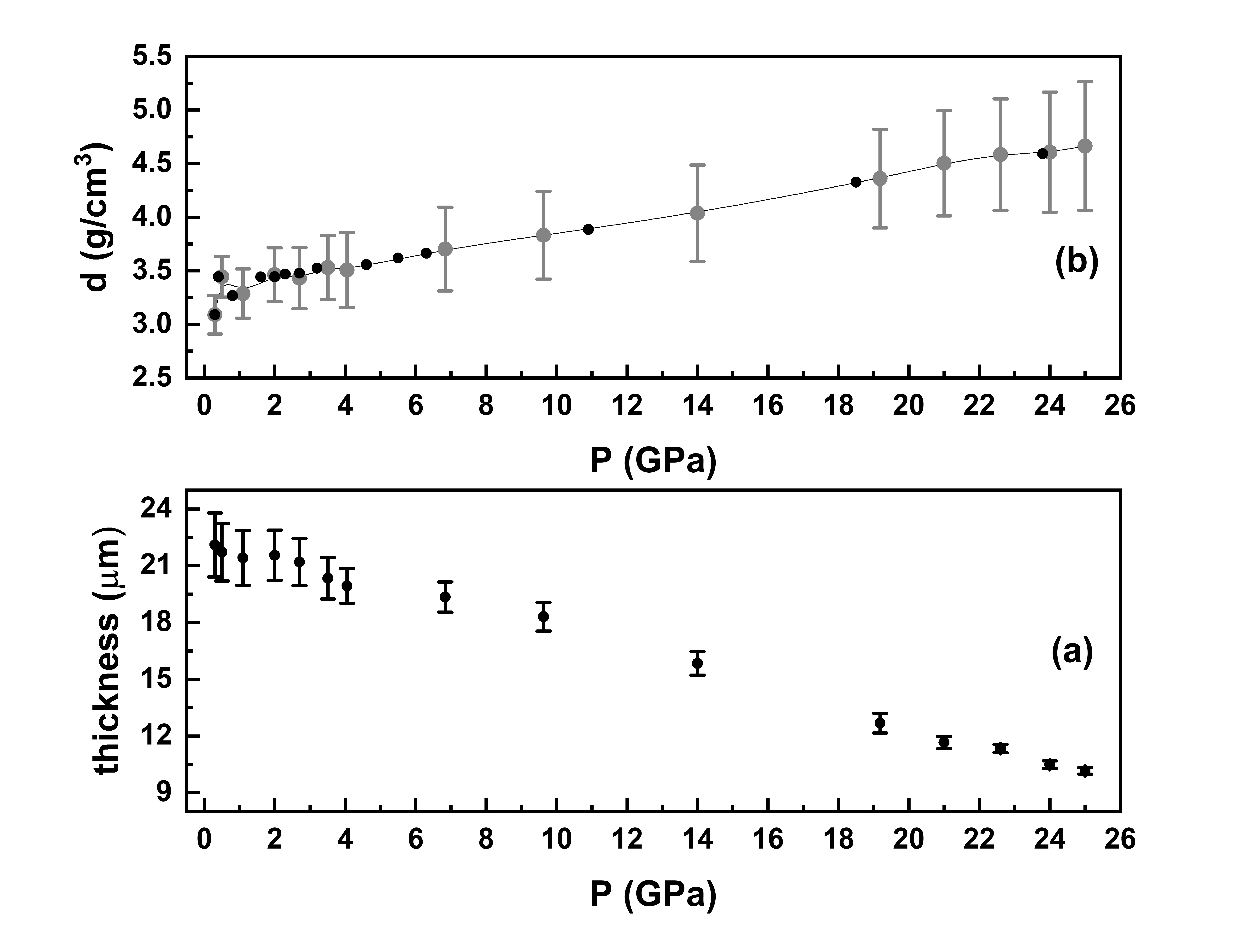
**

**References used in Supplementary Information**

[1]. Kalkan, B., Benmore, C. J., Aitken, B. G., Sen, S., Clark, S. M. A comparative study of the atomic structures of Ge-doped As4S3 and P4Se3 molecular glasses. *J. Non-Cryst. Solids* **514,** 83-89 (2019).

[2]. Krogh-Moe, J. A Method for Converting Experimental X-ray Intensities to an Absolute Scale. *Acta Crystallogr.* **9**, 951– 953 (1956).
